# Incidence and risk factor of allergic contact dermatitis to 2-octyl cyanoacrylate and n-butyl cyanoacrylate topical skin adhesives

**DOI:** 10.1038/s41598-021-03319-3

**Published:** 2021-12-09

**Authors:** Young Hwan Park, Jeong Seok Choi, Jung Woo Choi, Hak Jun Kim

**Affiliations:** Department of Orthopedic Surgery, Korea University Guro Hospital, 148 Gurodong-ro, Guro-gu, Seoul, 08308 Republic of Korea

**Keywords:** Medical research, Risk factors

## Abstract

Although the use of topical skin adhesives has increased as an alternative to conventional skin closure methods, studies on the incidence and risk factors of allergic contact dermatitis (ACD) to topical skin adhesives have been limited. The purpose of this study was to investigate the incidence and risk factors of ACD after the use of 2-octyl cyanoacrylate and n-butyl cyanoacrylate topical skin adhesives. We retrospectively reviewed 1145 patients (739 patients with 2-octyl cyanoacrylate and 406 patients with n-butyl cyanoacrylate) who underwent skin closure with topical skin adhesives. Variables suspected to correlate with ACD were retrieved from medical records and analyzed to determine risk factors. The incidence of ACD from the use of 2-octyl cyanoacrylate and n-butyl cyanoacrylate topical skin adhesives was 2.7% and 2.2%, respectively. There was no statistically significant difference in the incidence between the two ingredients. In logistic regression analysis, none of the variables were found to increase the risk of ACD in both 2-octyl cyanoacrylate and n-butyl cyanoacrylate topical skin adhesives. As ACD occurs without risk factors in 2–3% of patients who used 2-octyl cyanoacrylate or n-butyl cyanoacrylate topical skin adhesives, clinicians and patients should be aware of these facts before using topical skin adhesives.

## Introduction

As inappropriate skin closure can lead to cosmetically unacceptable scars, delayed healing, wound dehiscence, or infection, selection of skin closure method is important for orthopedic surgeons^1–3^. Over several decades, skin staples and sutures have been regarded as a common method for skin closure. However, despite their long period of usage, their fundamental shortcomings, such as time-consuming nature, cosmetic issues, and the need for removal are still unresolved^4–6^. For this reason, topical skin adhesives, which have the advantages of good scar cosmesis, infection prevention, easy skill acquisition, and lack of need for dedicated removal have begun to attract attention^7–13^.

Liquid 2-octyl cyanoacrylate and n-butyl cyanoacrylate monomers are commonly used ingredients for topical skin adhesives because they polymerize into solid chains upon contact with the wound surface and hold wound edges together^14^. Although 2-octyl cyanoacrylate and n-butyl cyanoacrylate are harmless to most patients, they can act as allergens and cause allergic contact dermatitis (ACD)^15,16^. ACD to topical skin adhesives usually presents as a self-limiting urticarial eruption near the incision site and subsides after proper local management. However, in some patients, it mimics postoperative infection, causes severe local or systemic reactions, and eventually, cosmetic sequelae^17^. Consequently, despite the advantages of topical skin adhesives, many orthopedic surgeons still hesitate to use them due to unpredictable adverse effects.

To date, ACD to topical skin adhesives has been continuously reported in case reports of sporadic events, but systematic studies on their incidence and risk factors have been limited. Therefore, this study aimed to investigate the incidence of ACD after the use of 2-octyl cyanoacrylate and n-butyl cyanoacrylate topical skin adhesives and to determine whether there are any risk factors for their occurrence.

## Patients and methods

After approval from the local ethics committee (Korea University Guro Hospital Institutional Review Board, No. 2021GR145), 1167 patients who underwent skin closure with topical skin adhesives in our institution between April 2017 and February 2021 were investigated for study eligibility. All patients underwent foot and ankle surgeries, which were performed by two orthopedic surgeons. Topical skin adhesives contained 2-octyl cyanoacrylate (Surgiseal, Adhezion Biomedical, Wyomissing, PA; Dermabond Prineo, Ethicon, Somerville, NJ) or n-butyl cyanoacrylate (LiquiBand, Advanced Medical Solutions, Winsford, UK). The exclusion criteria included age < 18 years, inflammatory disease (e.g., rheumatoid arthritis or psoriatic arthritis), active infection in the area of incision prior to surgery, or follow-up of less than 6 weeks. Twenty-two patients were excluded according to the criteria, resulting in the enrollment of 1145 patients in this study (195 patients using Surgiseal, 544 patients using Dermabond Prineo, and 406 patients using LiquiBand). All the study protocol were conducted in accordance with the relevant guidelines and regulations (STROBE guidelines)^18^. As a retrospective study, informed consent was waived by Korea University Guro Hospital Institutional Review Board.

After the surgical procedure of each patient, the fascia and subcutaneous layers were closed using 2–0 and 3–0 synthetic absorbable braided sutures. Topical skin adhesives were applied according to the instruction manual of each product. Surgiseal and LiquiBand were applied to two thin layers with a drying time of 30 to 60 s between layers to enable polymerization, and Dermabond Prineo was applied with the same process to a mesh. After that, a standard absorbent dressing was applied and changed on the second day after surgery. Postoperative dressing was performed with povidone-iodine, which was also used for preoperative skin preparation, at intervals of 2–3 days for up to 2 weeks after surgery.

**Figure 1:**
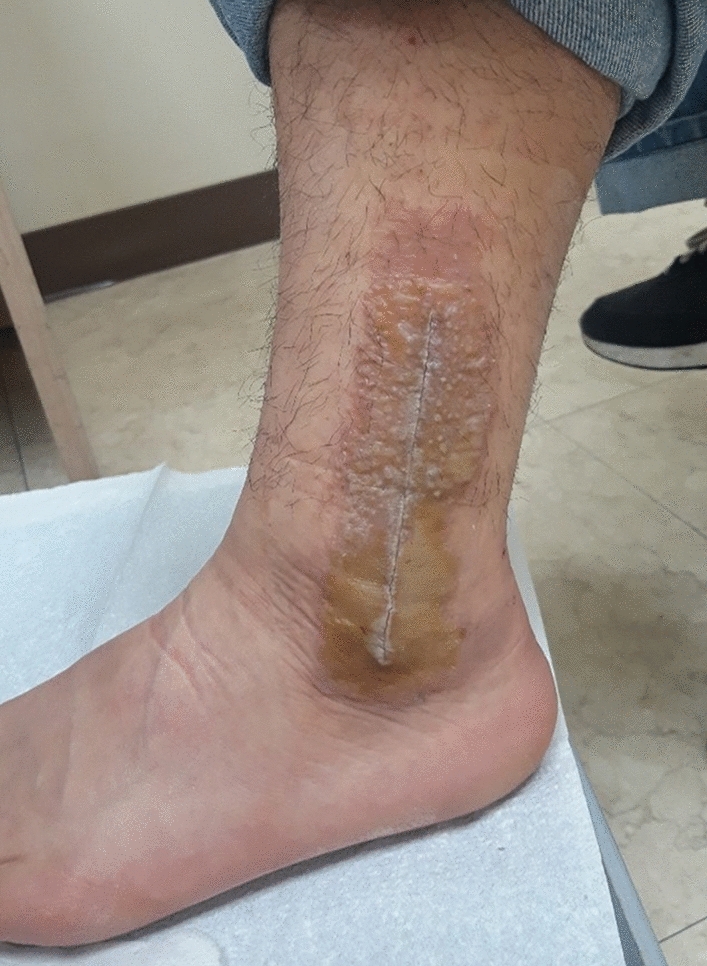
Clinical photograph of a patient showing well-demarcated pruritic eczematous eruption, papules, and vesicles that developed in the skin area to which topical skin adhesive was applied.

Patients were followed up between the first and second week after surgery, and surgical wounds were inspected by orthopedic surgeons. If the patients presented with a skin eruption that was not visible in ordinary cases, they were referred to a dermatologist for the diagnosis of ACD. The dermatologist diagnosed ACD based on history of exposure, morphology (well-demarcated pruritic eczematous eruptions, papules, or vesicles), and regional distribution (localized in the area of skin that was exposed to skin adhesives) (Fig. 1)^17,19^. The patch test was selectively performed when the lesions exhibited atypical features or a topical skin adhesive was not suspected to be the cause of dermatitis. ACD cases were treated using steroid ointment and oral antihistamines. If a superficial wound infection was suspected, oral antibiotics were administered. For wound dehiscence, adhesive tape was applied to the margins of the wound. None of the patients received systemic steroid therapy or wound revision.

The variables used to determine the risk factors of ACD to topical skin adhesives were retrieved from electronic medical records. As no definite factors related to the development of allergic contact dermatitis to topical skin adhesives are known, the variables that have been suspected to correlate with type IV hypersensitivity were included in the investigation, along with patient characteristics^20–22^. Two authors independently reviewed the medical records, and in case of any discrepancies between the two on the variables, a final decision was made through a consensus discussion.

### Statistical analysis

The difference in the incidence of ACD between ingredients or products was analyzed using the chi-square test. The risk factors for ACD were identified using logistic regression analysis of odds ratios. ACD was considered as a dependent variable, while age, sex, body mass index, diabetes, smoking, asthma, history of atopic dermatitis, history of contact dermatitis, neutrophil count, eosinophil count, and anatomical region were considered as independent variables. Continuous variables were analyzed with simple logistic regression, and categorical variables were analyzed using the chi-square test or Fisher exact test, as appropriate. Statistical analyses were performed using SPSS (version 23.0; IBM, Armonk, NY, USA), and significance was set at p-value < 0.05.

## Results

### Incidence of allergic contact dermatitis

Of the 1145 patients included in the study, 32 (2.8%) patients were referred to dermatologists for suspected ACD after the use of a topical skin adhesive in the foot and ankle surgery, and 29 (2.5%) patients were diagnosed with ACD. Of the three excluded patients, one had scabies and two had dyshidrotic eczema. Twenty of 739 (2.7%) patients using 2-octyl cyanoacrylate and nine of 406 (2.2%) patients using n-butyl cyanoacrylate showed ACD. Seven of 195 (3.6%) patients in Surgiseal, 13 of 544 (2.4%) patients in Dermabond Prineo, and nine of 406 (2.2%) patients in LiquiBand showed ACD. There was no difference in the incidence of ACD between ingredients or products.

### Risk factor for allergic contact dermatitis

None of the variables was found to increase the risk of ACD. Patient demographics are presented in Table 1. In the logistic regression analysis, age, sex, body mass index, diabetes, smoking, asthma, history of atopic dermatitis, history of contact dermatitis, neutrophil count, eosinophil count, and anatomical region did not show any correlation with the occurrence of allergic contact dermatitis (Table 2).

**Table 1:** Patient demographics.

Variable	2-octyl cyanoacrylate		n-butyl cyanoacrylate	
	Non-ACD (N = 719)	ACD (N = 20)	Non-ACD (N = 397)	ACD (N = 9)
Age, y	46.8 ± 16.2	41.5 ± 18.1	45.0 ± 16.7	37.9 ± 14.2
**Sex**				
Male	334	9	209	5
Female	385	11	188	4
**Diabetes**				
Yes	54	1	28	0
No	665	19	369	9
**Smoking**				
Yes	135	2	80	1
No	584	18	317	8
**Asthma**				
Yes	5	0	1	0
No	714	20	398	9
**History atopic dermatitis**				
Yes	11	0	5	0
No	708	20	396	9
**History of allergies**^**a**^				
Yea	36	1	24	1
No	683	19	373	8
Neutrophil count, %	56.5 ± 8.7	57.7 ± 9.2	56.8 ± 8.9	56.0 ± 10.1
Eosinophil count, %	2.7 ± 1.7	2.9 ± 2.1	2.7 ± 1.7	2.5 ± 0.7
**Anatomiccal region**				
Lower leg including ankle joint	333	12	192	7
Forefoot	131	3	54	1
Midfoot	114	2	47	1
Hindfoot	118	3	43	0

Data are mean ± standard deviation or number of patients (%).

*ACD* allergic contact dermatitis.

^a^Antigens other than topical skin adhesives.

**Table 2:** Results of logistic regression analysis to assess the risk factors of allergic contact dermatitis to topical skin adhesives.

Variable	ACD to 2-octyl cyanoacrylate (N = 20)		ACD to n-butyl cyanoacrylate (N = 9)	
	Odds ratio (95% CI)	p-value	Odds ratio (95% CI)	p-value
Age	0.981 (0.955–1.008)	0.159	0.229 (0.545–1.394)	0.547
Female	1.029 (0.362–2.926)	0.957	0.893 (0.201–3.979)	0.882
Diabetes	0.853 (0.107–6.822)	0.880	N/A	N/A
Smoking	0.469 (0.099–2.223)	0.340	0.397 (0.041–3.868)	0.427
Asthma	N/A	N/A	N/A	N/A
History of atopic dermatitis	N/A	N/A	N/A	N/A
History of allergies^a^	0.980 (0.116–8.319)	0.985	3.075 (0.280–33.761)	0.358
Neutrophil count	1.018 (0.967–1.071)	0.498	0.987 (0.915–1.065)	0.735
Eosinophil count	1.078 (0.843–1.379)	0.550	0.848 (0.531–1.353)	0.488
**Anatomiccal region**				
Lower leg including ankle joint	Reference value	0.806	Reference value	0.985
Forefoot	0.761 (0.205–2.824)	0.683	0.726 (0.082–6.410)	0.773
Midfoot	0.481 (0.105–2.199)	0.346	0.711(0.079–6.382)	0.761
Hindfoot	0.789 (0.212–2.929)	0.723	N/A	N/A

*ACD* allergic contact dermatitis, *CI* confidence interval, *N/A* not applicable.

^a^Antigens other than topical skin adhesives.

## Discussion

Although the use of topical skin adhesives has increased as an alternative to conventional skin closure methods, studies on the incidence and risk factors of ACD to topical skin adhesives have been limited. To address this issue, we reviewed 1027 patients who underwent skin closure with 2-octyl cyanoacrylate and n-butyl cyanoacrylate topical skin adhesives to investigate the incidence and risk factors of ACD to topical skin adhesives. The findings of this study showed that the incidence of ACD was 2.7% in 2-octyl cyanoacrylate and 2.2% in n-butyl cyanoacrylate, but none of the variables were found to increase the risk of ACD. In the trend of increasing the use of topical skin adhesives, these findings will help orthopedic surgeons attempt to use topical skin adhesives to predict ACD and provide information to patients.

Prior to this study, four studies have reported the incidence of ACD in the use of 2-octyl cyanoacrylate topical skin adhesive. Chalmers et al.^17^ investigated 6008 patients who used 2-octyl cyanoacrylate topical skin adhesive for elective orthopedic surgery and reported an incidence 0.5% of ACD. In addition, in joint arthroplasty surgery, Michalowitz et al.^23^ reported an incidence of 1.8% in 281 patients and Durando et al.^24^ reported 1.7% in 912 patients. Interestingly, Nakagawa et al.^25^ reported a 7.0% incidence of ACD in 100 patients who underwent breast reconstruction surgery. Except for Nakagawa et al., our incidence of 2.7% is somewhat higher than the previously reported incidence, but studies to date alone cannot clarify whether this is due to the difference in application site or patient demographics.

Unlike 2-octyl cyanoacrylate, to the best of our knowledge, there has been no study on the incidence of ACD in n-butyl cyanoacrylate topical skin adhesives. We deduced that this is due to its lower popularity than 2-octyl cyanoacrylate as a topical skin adhesive. Although the clinical studies showed equivalent results between 2-octyl cyanoacrylate and n-butyl cyanoacrylate topical skin adhesives^26,27^, the results of the animal studies showed that n-butyl cyanoacrylate is inferior in adhesion and wound-bursting strength than 2-octyl cyanoacrylate and would have led to less use of n-butyl cyanoacrylate than 2-octyl cyanoacrylate as a topical skin adhesive^28,29^. In fact, n-butyl cyanoacrylate is increasingly preferred for vascular embolization, bleeding hemostasis, or corneal injury repair over skin closure^30–32^. However, because n-butyl cyanoacrylate remains a viable option as a topical skin adhesive, the results of this study would be helpful for surgeons planning to use n-butyl cyanoacrylate topical skin adhesive.

Studies investigating the inherent risk factors of ACD found that females have a higher susceptibility to ACD than males and due to the difference in hormonal influences and exposure mode to the allergen^22^. However, in this study, the use of topical skin adhesive did not differ by sex, and the incidence of ACD was also not affected by sex. In addition, previous studies investigating the incidence of 2-octyl cyanoacrylate topical skin adhesives did not conclude that females are a risk factor for developing ACD^17,23,24^. Therefore, we thought that although females generally develop ACD more often than males, there is no sex difference in ACD that occurs after using 2-octyl cyanoacrylate and n-butyl cyanoacrylate topical skin adhesives. Similarly, when we analyzed the incidence of ACD in patients without risk factors for type IV hypersensitivity, as suggested in previous studies, the incidence of ACD was 2.5% (15/590) in 2-octyl cyanoacrylate and 2.2% (6/279) in n-butyl cyanoacrylate. As these values did not differ from those of all patients with possible risk factors (p = 0.857 and 0.955, respectively), we concluded that previously known risk factors for type IV hypersensitivity did not significantly affect the incidence of ACD in topical skin adhesives.

This study has two limitations. First, patch tests were not conducted on all suspected patients to diagnose ACD, so the accuracy of the diagnosis could be doubted. However, although the patch test can precisely identify the antigens, it is not an absolute diagnostic criterion for ACD and the clinical features of the patients were sufficient for dermatologists to diagnose ACD^19^. In addition, since the agent used for postoperative wound dressing was the same as that used for preoperative skin preparation, the area of the rash or erythema would have been more extensive than that of the topical skin adhesive if the patient was allergic to the dressing agent. Therefore, we believe that the presence or absence of a patch test would not have significantly affected the results of the study. Second, the patients in this study were confined to the application of topical skin adhesives to foot and ankle surgeries so that generalization of the results to the whole body can be questioned. However, considering that the mechanism of type IV hypersensitivity does not differ depending on the body part and the used site was not a factor affecting the occurrence of the ACD^17,22^, it is judged that the bias in the study population will not affect the generalization of the study results.

In conclusion, ACD, while uncommon, occurs in 2–3% of patients who used 2-octyl cyanoacrylate or n-butyl cyanoacrylate topical skin adhesives. ACD is difficult to predict due to the absence of risk factors, but clinicians and patients should be aware of these facts before using topical skin adhesives.

## Data Availability

All data supporting our findings are contained within the manuscript. All data in this study are freely available to any researcher for noncommercial purposes.

## Abbreviation

ACD: Allergic contact dermatitis
